# Hypertension intracrânienne idiopathique: à propos d’un cas rare associé à la grossesse

**DOI:** 10.11604/pamj.2017.27.143.12234

**Published:** 2017-06-28

**Authors:** Jihad Drissi, Ayman Hachi, Laila Adlani, Jaouad Kouach, Driss Moussaoui, Mohamed Dehayni

**Affiliations:** 1 Service de Gynécologie-Obstétrique, Hôpital Militaire d’Instruction Mohamed V, Rabat, Maroc

**Keywords:** Hypertension intra-crânienne, idiopathique, grossesse, pronostic visuel, shunt, Idiopathic intracranial hypertension, pregnancy, visual prognosis, shunt

## Abstract

Nous rapportons le cas d’une primigeste de 25ans dont la grossesse a été compliquée, dès le premier trimestre, d’une hypertension intracrânienne (HTIC) idiopathique avec retentissement visuel, ayant bénéficiée d’un shunt lombo-péritonéal sans conséquences obstétricales. L’objectif de ce travail est de préciser les caractéristiques de cette entité pathologique rare dont le mécanisme physiopathologique est mal élucidé. Elle serait liée à un défaut de réabsorption du liquide céphalorachidien (LCR) au niveau des granulations arachnoïdiennes. Les principaux facteurs de risque évoqués sont : l’obésité, le syndrome des ovaires polykystiques, la thrombophilie et l’hyperfibrinolyse. Le diagnostic repose sur les critères modifiés de Dandy après enquête clinico-biologique et radiologique négative. Comme pour l’HTIC « classique », le pronostic visuel est compromis. Cependant, il n’y a pas de risque d’engagement qui mettrait en jeu le pronostic vital. Par ailleurs, cette maladie n’influence pas l’évolution de la grossesse. Ceci dit, un traitement rapide et efficace devra être instauré afin de préserver la fonction visuelle de ces patientes.

## Abstract (en)

We report the case of a 25-year old primipara whose pregnancy was complicated by idiopathic intracranial hypertension (ICHT) associated with visual impairment in the first quarter. She underwent lumboperitoneal shunt without obstetric consequences. This study aimed to determine the features of this rare pathological entity whose pathophysiological mechanism is poorly elucidated. It would be caused by poor absorption of cerebrospinal fluid (CSF) through the arachnoid granulations. Major risk factors are: obesity, polycystic ovary syndrome, thrombophilia and hyperfibrinolyse. Diagnosis is based on modified Dandy criteria after negative clinico-biological and radiological assessment. Visual prognosis is compromised, as in the case of « classical » ICHT. However, there is no risk for cerebral involvements which could be life-threatening. In addition, this disease does not influence pregnancy outcome. This said, rapid and effective treatment should be implemented in order to preserve visual function in these patients.

## Introduction

Décrite pour la première fois par Quicke en 1897, l’HTIC bénigne, appelée également HTIC idiopathique ou pseudotumeur cérébrale, est une augmentation de la pression du liquide céphalorachidien (LCR) sans arguments cliniques, biologiques ou radiologiques en faveur d’une pathologie intra-crânienne [1, 2]. Nous rapportons le cas d’une patiente ayant présenté une HTIC idiopathique diagnostiquée au premier trimestre de sa grossesse. L’objectif de ca travail est de préciser les particularités de prise en charge de ces patientes en mettant le point sur les conséquences obstétricales de cette pathologie.

## Patient et observation

Mme S. âgée de 25ans, enceinte de 38 semaines d’aménorrhée (SA), sans antécédents pathologiques notables notamment pas d’antécédents de thrombophilie ou de maladie de système, avec un indice de masse corporel de 23kg/m^2^. Décrivant la survenu brutale à 13SA d’un syndrôme d’HTIC fait de céphalées en casque, vomissements et troubles visuels, sans syndrôme méningée et sans signes de localisation. L’IRM (imagerie par résonnance magnétique) cérébrale n’a trouvée aucun signe en faveur d’une hydrocéphalie, d’œdème cérébral, ou de processus expansif intra-crânien. L’examen ophtalmologique a objectivé une altération bilatérale marquée du champ visuel. La ponction lombaire (PL) a permis de confirmer l’hyperpression intra-crânienne, de soulager la patiente et de réaliser l’analyse biologique du LCR revenue normale, permettant ainsi d’affirmer le diagnostic. Aussi, un traitement à base d’acétazolamide 1000 puis 1500mg/j visant à réduire la production du LCR par les plexus choroïdes a été instauré avec cependant la récurence des épisodes d’HTIC d’où la réalisation d’une dérivation du LCR par shunt lombo-péritonéal. Le déroulement de la grossesse était sans anomalies ; la morphologie et la croissance fœtale étaient correctes.

La patiente a été admise à 38SA en dehors du travail pour rupture prématurée des membranes remontant à 10heures, sans signes d’HTIC à l’examen clinique. L’échographie obstétricale a objectivé un retard de croissance intra-utérin modéré sans inversion du rapport cérébroplacentaire en vellocimétrie doppler. Devant le contexte d’HTIC idiopathique engageant le pronostic visuel, une césarienne extrapéritonéale sous rachi-anesthésie a été réalisée, donnant naissance à un nouveau-né à terme de 2150g ayant un score d’apgar à 10/10. Les suites opératoires étaient simples pour la mère et le nouveau-né. Un contrôle ophtalmologique (fond d’œil et champ visuel) et un bilan de thrombophilie sont prévus. Cette situation pathologique ne contre-indique nullement une éventuelle grossesse ultérieure vu le risque faible de récidive.

## Discussion

L’HTIC idiopathique est une entité rare, dont l’incidence est estimée à 1/100 000/an dans la population générale, avec une atteinte préférentielle des femmes âgées de 20-44ans (19/100 000) [1, 3]. Liée aux situations associées à une altération de la réabsorption du LCR. Il s’agit d’une complication exceptionnelle de la grossesse dont l’incidence chez les femmes enceintes n’excède pas 1/870. Elle peut survenir aux trois trimestres de la gestation et semble être favorisée principalement par l’obésité, le syndrome des ovaires polykystiques, la thrombophilie et l’hyperfibrinolyse [1–3]. Ces facteurs interfèrent avec les mécanismes de régulation de la pression du LCR et de la circulation veineuse et artérielle [4].

Le mécanisme physiopathologique précis est mal élucidé. Plusieurs théories ont été avancées. Cependant il est actuellement admis que cette pathologie est en rapport avec un défaut de réabsorption du LCS au niveau des granulations arachnoïdiennes avec une implication probable du système veineux qui serait la voie finale commune des théories physiopathologiques. En effet, il a été constaté que 9.4% des cas d’HTIC bénignes étaient associés à une thrombose d’un sinus dural ou à une sténose d’un sinus veineux [2, 5].

Le diagnostic d’HTIC idiopathique lié à la grossesse devra être évoqué chez toute femme enceinte présentant un syndrôme d’HTIC avec un bilan étiologique exhaustif normal. En effet, il s’agit d’un diagnostic d’exclusion qui sera retenu si tous les critères modifiés de Dandy sont remplis [6]: 1) Signes d’HTIC (céphalées, nausées, vomissements, œdème papillaire, éclipse visuel). 2) Examen neurologique normal excepté pour la paralysie du IV. 3) Elévation de la pression du LCR à plus de 20cmH2O (plus de 25cmHg chez le sujet obèse). 4) Composition normale du LCR. 5) Neuro-imagerie montrant des petits ventricules symétriques et excluant un syndrôme de masse ou toute autre cause d’élévation de la pression intra-crânienne (PIC).

Le diagnostic chez notre patiente a été retenu se référant à ces critères de diagnostic. En effet, elle a présenté un syndrome d’HTIC , sans syndrome méningée et sans signes de focalisation à l’examen neurologique. La ponction lombaire a permis d’objectiver l’hyperpression du LCR et d’éfectuer une analyse cytobactériologique et biochimique revenue normale. L’IRM (imagerie par résonnance magnétique) cérébrale n’a trouvée aucun signe en faveur d’une hydrocéphalie, d’un œdème cérébral, ou d’un processus expansif intra-crânien.

La PIC peut être mesurée soit au cours d’une PL, soit par méthodes invasives par capteurs intra-parenchymateux. Dans tous les cas un enregistrement continu est souhaitable (histogramme sur 15-30min) [1]. L’imagerie des sinus veineux joue un rôle de plus en plus important dans l’évaluation de l’HTIC bénigne car une gêne du retour veineux est fréquemment retrouvée, l’angio-IRM convient parfaitement à cette situation elle recherchera une thrombose ou une sténose veineuse sinusienne [7].

L’évolution de la grossesse ne semble pas être influencée par la maladie, sauf dans l’expérience de Huna-Baron et Kupersmith qui ont relevés deux MFIU sur 16cas. L’acétazolamide devra être évité avant 20SA puisqu’un cas de tératome sacro-coccygien a été rapporté après administration du traitement. Contrairement au cas d’HTIC « classique » l’HTIC idiopathique n’est pas associée au risque d’engagement qui mettrait en jeu du pronostic vital. Cependant, le pronostic visuel est engagé avec risque de cécité définitive faisant toute la gravité de cette pathologie neurologique [**2**]. Chez notre patiente aucun retentissement obstétrical n’a été noté. Néanmoins les poussées répétitives d’HTIC se sont compliquées d’ une amputation marquée du champs visuel .

Il s’agit d’une urgence thérapeutique dont la prise en charge diffère peu de celle des autres types d’HTIC, elle repose sur: 1) Les mesures hygiéno-diététiques. En effet, une réduction pondérale de 35kg est associée à une baisse moyenne de la PIC de l’ordre de 19cmH2O. 2) Les PL itératives. 3) L’instauration d’une corticothérapie ne franchissant pas la barrière placentaire, à base de prednosone, à la dose de 40-60mg/j. 4) Afin de réduire la production du LCR par les plexus choroïdes l’acétazolamide, infructueux chez notre patiente, est prescrit. Ce médicament est compatible avec l’allaitement selon l’Académie Américaine de Pédiatrie. En cas d’intolérance il sera remplacé par un diurétique de l’anse (le furosémide) à la dose maximale de 40mg trois fois par jour pour une durée limitée vu le risque de déshydratation [2, 8, 9].

En cas d’échec de ces thérapeutiques, la prise en charge chirurgicale sera indiquée particulièrement dans les formes malignes compromettant le pronostic visuel, ce qui a été le cas de notre patiente, qui malgré la prise d’acétozolamide a continué à présenter des pics d’HTIC compromettant considérablement son pronostic visuel. Le traitement chirurgicale consiste en une fenestration de la gaine du nerf optique avec dérivation du LCR par un shunt lombo-péritonéal ou ventriculo-péritonéal qui permet de décomprimer le nerf optique et d’améliorer la vision dans 75% des cas dans les 3à 4semaines. Récemment la mise en place d’une endoprothèse sinusienne durale par voie endovasculaire a été proposée dans certains cas de sténose veineuse sinusienne [10, 11].

En dehors de tout contexte de poussée d’HTIC l’accouchement par voie basse n’est pas contre-indiqué, l’anesthésie péridurale est recommandée de même que les extractions instrumentales afin de réduire les efforts expulsifs. La césarienne sera réservée aux indications obstétricales, elle sera réalisée sous anesthésie loco-régionale malgré le risque de lésion du shunt lombo-péritonéale. En effet, l’anesthésie générale est à éviter en raison du risque des troubles ventilatoires chez ces patientes souvent obèses [2]. Le risque de récurrence au cours des prochaines grossesses est faible ; seuls quelques cas de récidive dans un contexte d’obésité ou de drépanocytose ont été rapportés dans la littérature [2].

## Conclusion

Quoique rare, l’HTIC idiopathique doit être évoquée chez toute femme enceinte présentant un syndrome d’HTIC sans étiologie évidente. Le diagnostic repose sur les critères modifiés de Dandy. Il s’agit d’une urgence médico-chirurgicale. Cette neuropathie ne retentit pas sur l’évolution de la grossesse. L’accouchement par voie basse n’est pas contre-indiqué dans les formes contrôlées en dehors de tout contexte de poussée d’HTIC. En post-partum un bilan de thrombophilie devrait être réalisé. Le risque de récidive si une nouvelle grossesse venait à être envisagée est faible.

## Conflits d’intérêts

Les auteurs ne déclarent aucun conflit d'interêt.

## References

[1] Chazal J, Klein O (2008). Hypertension intracrânienne bénigne: historique, définition et physiopathologie. Neurochirurgie..

[2] Jacopin-Bruneau L, Gommier B, Pierre F, Boog G (2010). Hypertension intracrânienne bénigne et grossesse - À propos de deux cas. Journal de Gynécologie Obstétrique et Biologie de la Reproduction..

[3] Glueck CJ, Iyengar S, Goldenberg N, Smith LS, Wang P (2003). Idiopathic intracranial hypertension: associations with coagulation disorders and polykystic-ovary syndrome. J Lab Clin Med..

[4] Biousse V, Bousser MG (2001). L’hypertension intracrânienne bénigne. Rev Neurol..

[5] Klein O, Joud A, Marchal J-C (2008). Prise en charge de l’hypertension intra-crânienne bénigne : analyse de la série nancéenne. Neurochirurgie..

[6] Riss I (2005). OEdème papillaire dans l’hypertension intracrânienne idiopathique. EMC-Neurologie..

[7] Bracard S (2008). Hypertension intracrânienne bénigne: imagerie et thérapeutiques endovasculaires. Neurochorurgie..

[8] Dhellemmes P, Defoort S, Vinchon M (2008). Hypertension intracrânienne bénigne: place du traitement médical. Neurochirurgie..

[9] Erguig L, Benomar A (2004). Hypertension intracrânienne bénigne - Aspects cliniques et thérapeutiques. Revue Neurologique..

[10] Metellus P (2007). Traitement endovasculaire de l’hypertension intracrânienne bénigne étiqueté « idiopathique » - Analyse de 8 cas consécutifs. Neurochirurgie..

[11] Metellus PH (2005). Traitement endovasculaire d’un syndrome d’hypertension intracrânienne bénigne par mise en place d’un stent dans le sinus latéral: considérations thérapeutiques et physiopathologiques à propos d’un cas. Neurochirurgie..

